# “Take It or Leave It”—Factors Regulating Competence Development and DNA Uptake in *Campylobacter jejuni*

**DOI:** 10.3390/ijms221810169

**Published:** 2021-09-21

**Authors:** Julia C. Golz, Kerstin Stingl

**Affiliations:** National Reference Laboratory for Campylobacter, Department of Biological Safety, German Federal Institute for Risk Assessment, 12277 Berlin, Germany; Julia.Golz@bfr.bund.de

**Keywords:** natural transformation, genetic diversity, adaptation, pH regulation

## Abstract

*Campylobacter jejuni* has a large adaptive potential due to enormous genetic exchange. Factors regulating natural transformation in this food-borne pathogen are largely unknown but of interest for the application of sustained reduction strategies in the food-processing industry. Using a single cell DNA uptake assay, we visualized that recognition of methylated *C. jejuni* DNA was essential for the first step of DNA uptake into a DNase resistant state. Transformation rates using a resistance marker correlated with the fraction of competent bacteria, harboring one to maximally four locations of active DNA uptake, not necessarily being located at the cell pole. Competence developed with rising pH between 6.5 and 7.5 under microaerobic conditions and was nearly insensitive towards growth temperatures between 32 °C and 42 °C, CO_2_ concentrations ranging from 0 to 50% and growth rates. However, competence development was abolished at pH 5 or under aerobic stress conditions, in which the bacteria ceased growth but fully survived. The DNA uptake machinery in competent bacteria shut down at slightly acidic pH and was reversibly switched on upon neutralization. It was dependent on the proton motive force and, in contrast to competence development, slightly enhanced under aerobic conditions. The results suggest that natural transformation in *C. jejuni* occurs in the neutral and microaerobic intestinal environment for enhanced genetic diversity and pre-adaption before host switch. In addition, highly competent bacteria might be shed into the environment, still able to acquire genetic material for increased survival.

## 1. Introduction

*Campylobacter jejuni* is a human foodborne pathogen causing the majority of bacterially induced gastroenteritis in the European Union, with 220,682 reported cases in 2019 [1]. The pathogen resides in birds, but also pigs, cattle and other animals are frequently colonized by the bacterium [2,3,4]. *C. jejuni* infection of humans primarily occurs via contaminated food, in particular due to cross-contamination from raw poultry meat to ready-to-eat fresh food and by consumption of undercooked poultry, other raw meat and milk [5,6].

*Campylobacter* is highly sensitive to environmental stress. Microaerobic conditions and temperatures between 37 °C and 42 °C are optimal growth conditions for *C. jejuni*, while the bacterium cannot grow below 30 °C or under atmospheric oxygen levels [7,8]. However, *Campylobacter* exhibits enormous survival strategies and is highly adapted to surviving harsh conditions by undergoing the viable but non-culturable state [9], but also by adaptation mechanisms [10,11,12] and by genetic exchange [13]. Recently, we identified by whole genome sequencing with *k*-mer analysis, that some *C. coli* strains harbored 15% introgression of *C. jejuni* sequences [14]. The gene set with *C. jejuni* introgression displayed potential roles in stress defense, suggesting enormous adaptation by natural transformation.

Although natural transformation is widespread among bacteria and offers the chance to acquire new genes or gene variants, it may be harmful for genome integrity [15,16,17]. Therefore, the process is usually regulated and limited to a subset of the bacterial population. Bistability of bacteria, tightly regulated using a main competence regulator or silencing of DNA uptake genes by non-coding small RNA have been identified to integrate various internal and external stimuli for the control of competence development and DNA uptake in some example bacteria, like *Bacillus subtilis*, *Streptococcus*, *Vibrio* and *Legionella* [18,19,20]. However, for most other bacteria, even the parameters for stimulating or inhibiting competence development are largely unknown.

The expression of the competence genes encoding a type IV secretion system in a close relative of *C. jejuni*, *Helicobacter pylori,* was differently expressed in a strain-dependent manner [21]. Natural transformation in *H. pylori* was shown to be tightly regulated by pH and oxidative stress [22]. A pH above 6.5 opened up a window for transformation, in which increase in pH and oxygen levels drastically stimulated DNA uptake, conditions prevailing at the multiplication site of the pathogen. In *Campylobacter*, natural transformation was observed to be most effective in exponential growth phase and parameters were, thereafter, varied within a 4 h assay with contact to chromosomal DNA and selection of resistant transformants [23]. In that study alkaline pH, 42 °C and aerobic as well as microaerobic conditions led to increase in the number of transformants. In another study, a positive correlation between lower CO_2_ concentrations and higher transformation levels was described [24]. A growth condition, in which *C. jejuni* did not take up any DNA was not identified. 

In contrast to *H. pylori*, *C. jejuni* also restricts DNA uptake by differentiation between own and foreign external DNA [25] by recognition of a CtsM methylated RATTY motif frequently present in *C. jejuni* chromosomal DNA [26]. The mechanism of DNA uptake in *C. jejuni* is similar to other bacteria, using a type II-secretion/type IV pilus system in combination with the ComEC master inner membrane channel, which is common to all transformable bacteria [27,28,29,30].

Here we used a single cell-based assay to directly monitor DNA uptake in the foodborne pathogen *C. jejuni*. For the first time we were able to independently test parameters for stimulating or inhibiting competence development or DNA uptake. As observed for *H. pylori*, competence development was dependent on neutral to slightly alkaline pH. In contrast, aerobic conditions prevented competence development but not DNA uptake. Interestingly, *C. jejuni* grew in the absence of CO_2_ despite of increased generation times, with competence development being unaffected. The DNA uptake itself was energy-dependent, insensitive against oxygen but shut down at slightly acidic pH. More close understanding of the mechanisms and the parameters influencing natural transformation in *C. jejuni* are useful for the development of strategies inhibiting the pathogen’s adaptive potential. This may support the design of sustainably effective reduction strategies against the pathogen in food-supplying animals. 

## 2. Results

### 2.1. DNA Uptake in C. jejuni Occurs at Distinct Locations, Is DNA Substrate Specific and Energy-Dependent

**Figure 1:**
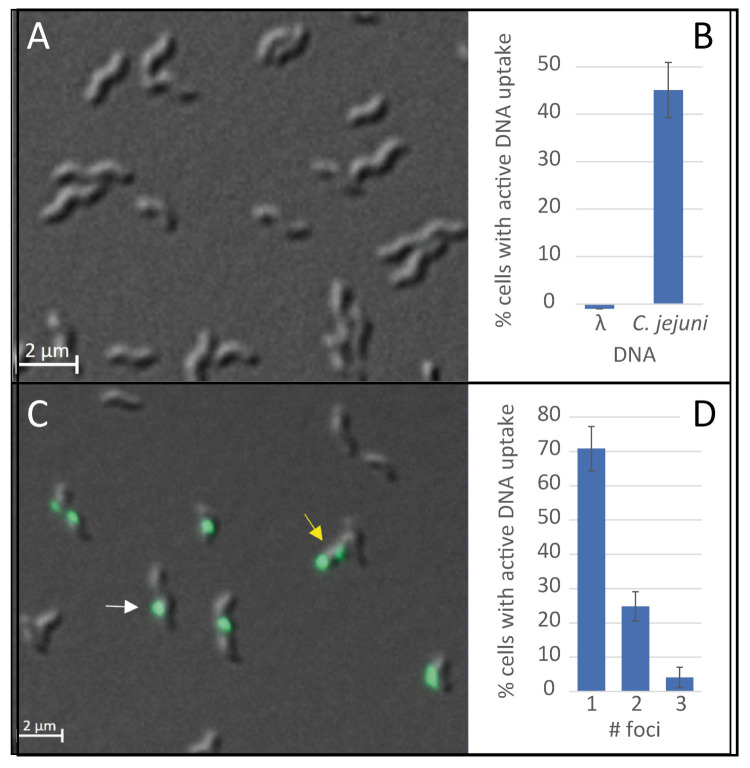
DNA uptake in *C. jejuni* BfR-CA-14430 occurs at distinct locations and is dependent on the DNA substrate. *C. jejuni* were incubated with fluorescently labelled DNA of the bacteriophage λ (**A**) or *C. jejuni* DNA (**C**) and the fraction of cells with active DNA uptake (**B**) and the distribution of number of DNA foci (**D**) after contact to *C. jejuni* DNA were quantified. Arrows in (**C**) indicate example bacteria with one (white) and two (yellow) DNA uptake locations. Overlay image of differential phase contrast (DIC) and Fluorescein channel. Scale bar, 2 µm. Experiments were performed at least three times. Error bars indicate standard deviation. #, number.

In contrast to *H. pylori*, *C. jejuni* DNA uptake is limited to DNA substrates, which harbor RATTY sites methylated by *Campylobacter* spp. specific CtsM methylase [26]. Thus, in order to establish the single cell assay for *C. jejuni*, genomic DNA extracted from *C. jejuni* served as DNA substrate, rather than DNA of the *E. coli* bacteriophage λ, previously used for *H. pylori*. As proof-of-principle, both DNA substrates were labelled with fluorescein and *C. jejuni* cells in exponential growth phase were challenged by these two sorts of covalently labeled DNA. After incubation at 37 °C for 30 min and subsequent degradation of external non-incorporated DNA by 5 min of DNaseI digestion at 37 °C, bacteria were immobilized using an agarose gel surface and microscopically analyzed. The bacteria harboring at least one DNase-resistant fluorescent focus were considered competent for natural transformation (Figure 1). Using λ-DNA lacking methylation at the recognition motif RATTY, no DNA focus was detected in *C. jejuni* BfR-CA-14430 grown for 18 ± 4 h in Bolton broth (final pH of 7.5) (Figure 1A,B). When *C. jejuni* genomic DNA was used as substrate, bacteria showed competence levels of around 45.1 ± 5.8% (Figure 1B,C). Analyzing this competent fraction in detail, 70.8 ± 6.5% of the competent cells harbored one DNase-resistant fluorescent focus, 24.8 ± 4.3% showed two foci and 4.1 ± 3.0% even displayed three visually separated locations of DNA uptake (Figure 1D). Only very few bacteria were observed with four DNA foci (0.3 ± 0.3%). 

This result suggests that recognition of methylated RATTY is necessary at the very first step of DNA uptake, namely transport of DNA from the environment into a DNase-resistant state (periplasm). It also corroborates the idea that the assay was suitable for detection of DNA uptake into *C. jejuni*. 

**Figure 2:**
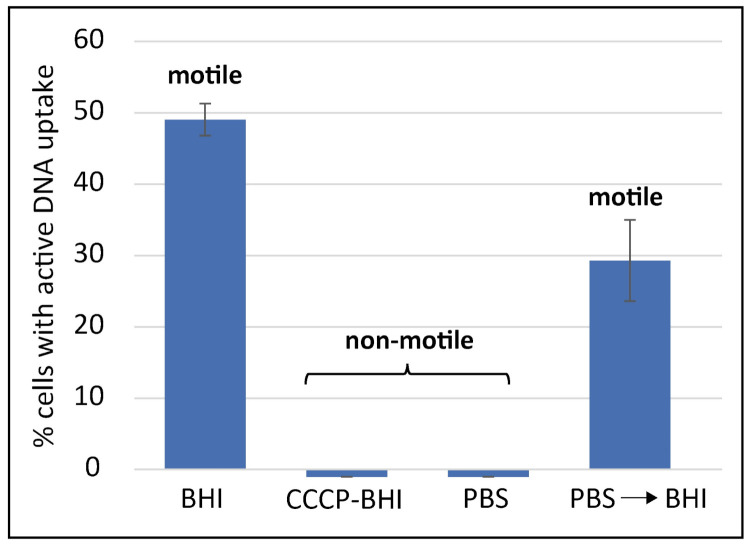
DNA uptake requires proton motive force. Competent *C. jejuni* pre-incubated for 10 min in the presence of the protonophore CCCP or nutrient-depleted for 2 h in PBS showed abolished DNA uptake capacity and motility. Re-energized cells (PBS→BHI; 2 h in PBS shifted to BHI during DNA uptake) regained motility and the ability to take up DNA. Experiments were performed at least three times. Error bars indicate standard deviation. PBS, phosphate-buffered saline; BHI, brain heart infusion; CCCP, carbonyl cyanide m-chlorophenyl hydrazine.

We further checked if the process of DNA uptake was dependent on the proton motive force (pmf), as shown for other bacteria [31,32]. For this purpose, we modulated the conditions during the time period, in which competent cells were in contact with the fluorescent genomic *C. jejuni* DNA. Under “standard DNA uptake conditions” using brain heart infusion (BHI), nearly half of the bacteria (49.1 ± 2.2%) showed active DNA uptake within 30 min of uptake period (Figure 2). When *C. jejuni* were pre-incubated for 10 min with 250 µM of the protonophore, carbonyl cyanide m-chlorophenyl hydrazine (CCCP), which abolishes the proton gradient across the inner membrane [33], DNA uptake capacity was completely lost (Figure 2). 

Furthermore, we limited the nutrient supply by incubation of competent cells for 2 h in phosphate-buffered saline (PBS) under microaerobic conditions. Under these conditions, the bacteria were non-motile, as also noticed in the presence of CCCP, indicating that pmf is limiting, which drives flagellar movement [34]. Those nutrient-depleted *C. jejuni* were subsequently incubated with fluorescent DNA in the presence of PBS or in re-energizing BHI. While *C. jejuni* were not able to take up DNA in PBS, 29.3 ± 5.7% of the bacteria in BHI took up DNA into a DNase resistant state (Figure 2). These re-energized bacteria also regained motility under this condition. We conclude that *C. jejuni* requires the proton motive force for DNA uptake. It also indicated that uptake is favored under nutrient-rich conditions. Consistently, DNA uptake performed under aerobic conditions led to a 1.28 ± 0.26-fold increased fraction of bacteria with a visible DNA focus than under microaerobic conditions (*n* = 20; Figure S1). 

### 2.2. Competence Development in C. jejuni Is Dependent on External pH and Shut Down under Aerobic Conditions

**Figure 3:**
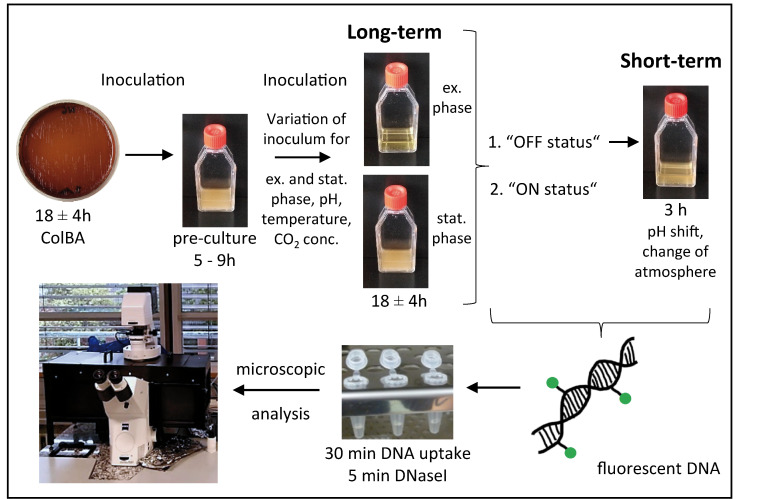
Schematic overview of the experimental setup of the single cell assay. All cells grown on ColBA plates were pre-cultured in Bolton broth for 5–9 h to stationary phase. A subculture was prepared with variation of inoculum (to reach exponential or stationary phase), pH, temperature, CO_2_ concentration and incubated microaerobically for 18 ± 4 h. These cells were analyzed in the framework of the “long-term” setup. Cultures (from the “long-term” setup) in “OFF status” were challenged for 3 h by “short-term” pH elevation and/or atmospheric shift. After adding fluorescently labelled DNA and subsequent DNaseI digestion, bacteria were analyzed by fluorescence microscopy. Ex. phase, exponential phase culture with OD_600nm_ of 0.1–0.5; stat. phase, stationary phase culture with OD_600nm_ of 0.8–1.5; conc., concentration.

In order to decipher parameters regulating competence development in *C. jejuni* we used a distinct experimental setup, in which we monitored “long-term” and “short-term” effects on competence development, such as growth phase, pH, temperature, oxidative stress and CO_2_ concentration (Figure 3). For fastidious bacteria, it is important to adapt optimal growth conditions in order to obtain physiological relevant data. For this purpose, *C. jejuni* were pre-cultured from a fresh 18 ± 4 h culture on Columbia blood agar (ColBA) at an initial OD_600nm_ of around 0.3 in liquid Bolton broth basis (without selective supplements) for 5 to 9 h to OD_600nm_ of ~1.5 before sub-culturing the bacteria in the same medium. The sub-culture was modified with respect to inoculum so that *C. jejuni* reached either exponential (OD_600nm_ of 0.1 to 0.5) or stationary growth phase (OD_600nm_ of 0.8 to 1.5) after 18 ± 4 h of incubation (“long-term” setup). Therefore, very low initial OD_600nm_ between 3 × 10^−7^ and 2.5 × 10^−5^ for exponential or 1–5 × 10^−3^ for stationary phase cultures were inoculated. In Bolton broth at pH 7.5 growth rate of *C. jejuni* was highly reproducible with a mean generation time of 1.2 ± 0.1 h (exponential phase, Table S1). To determine the effect of pH on competence development, pH of the medium was adjusted with sodium hydroxide (NaOH) or hydrochloric acid (HCl). 

**Figure 4:**
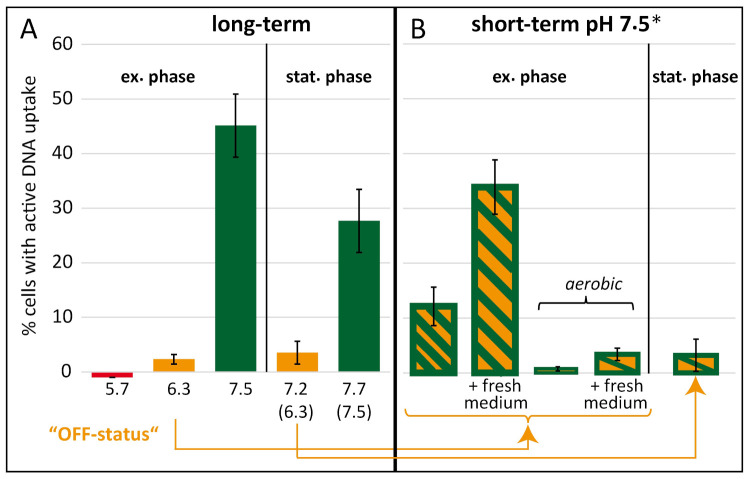
pH and atmosphere determine competence development in *C. jejuni*. Experimental setups for “long-term pH” (**A**) and “short-term pH shift” (**B**) of *C. jejuni* BfR-CA-14430 were as described in Figure 3. Final pH values ± 0.2 of cultures tested in the DNA uptake assay were as indicated, with initial pH-values ± 0.2 of original (exponential) cultures in brackets. The exponential culture with a final pH 6.3 and its corresponding stationary culture without pH adjustment (pH 7.2) were used as “OFF-status” cultures for short-term pH shifts. Ex. phase, exponential phase culture with OD_600nm_ of 0.1–0.5; stat. phase, stationary phase culture with OD_600nm_ of 0.8–1.5. Fresh medium, cultures were diluted 3 to 5-fold with Bolton broth; aerobic, atmosphere was changed to aerobic conditions for short-term challenge; *, final pH 7.9 instead of 7.5 due to additional release of CO_2_ from the medium. DNA uptake occurred under aerobic conditions at pH 7.5 ± 0.1 except for culture pH 5.7, for which uptake was kept at the same pH value of 5.7 (see also Table 1). Experiments were performed at least three times. Error bars indicate standard deviation.

Indicated pH values in the text and in Figure 4 are final pH values after growth ± 0.2. The titrated pH at standard microaerobic conditions did not change during growth from inoculation until exponential growth phase (Figure S2, yellow squares) but was slightly increased in stationary growth phase (Figure 4, pH values in brackets) due to *C. jejuni* metabolic activity at higher cell densities. The fraction of competent bacteria in exponential phase at pH 7.5 was 45.1 ± 5.8% (Figure 4). In stationary growth phase at a final pH of 7.7 this fraction of bacteria was lower comprising 27.7 ± 5.8%. When the pH was lowered to 6.3, a drastic decrease of the fraction of competent bacteria was observed in exponential growth phase, since only 2.3 ± 0.9% of bacteria were observed with at least one fluorescent DNA focus (Figure 4A). A parallel culture with initial pH of 6.3 but grown to stationary growth phase was likewise only marginally competent with 3.5 ± 2.1% of bacteria (Figure 4A). For all conditions depicted in Figure 4, the incubation of bacteria with fluorescent DNA was done at pH 7.5. An exception to this rule was the culture at pH 5.7, which was challenged with DNA at the same pH of 5.7 in order to see, if DNA uptake in *C. jejuni* could be completely abolished under these conditions. Indeed, not a single bacterium was observed to take up fluorescent DNA under these conditions, suggesting that slight acidic pH is sufficient for complete shut-down of natural transformation in *C. jejuni*. However, we chose the exponential pH 6.3 and the corresponding stationary culture at 7.2 as “OFF-status” cultures for the “short-term” setup (as indicated in Figure 4A), since the generation time of *C. jejuni* at pH 5.7 was considerably extended (Table S1).

“OFF-status” bacteria were challenged by a pH shift to 7.5 for 3 h in the “short-term” setup (Figure 4B). When the pH upshift was established by direct titration of the culture with NaOH, 12.2 ± 3.5% of the cells took up fluorescently labelled DNA. In case we upshifted the exponential phase culture by 3- to 5-fold dilution in fresh Bolton broth at pH 7.5 to OD_600nm_ 0.05, even 33.9 ± 5.0% of the bacteria displayed active DNA uptake capacity (Figure 4B), indicating that fresh medium stimulated competence development. The stationary “OFF-status” culture did not respond during the 3 h, indicating that those cells lost capacity to switch into the competent phase (Figure 4B). 

Interestingly, when the exponential “OFF-status” culture was pH-upshifted under aerobic conditions, competence levels even decreased to 0.8 ± 0.4% or were stable in the presence of fresh medium at low levels (Figure 4B). Since under aerobic conditions, *C. jejuni* is stressed by high oxygen levels and does not grow, survival of the bacterium under these conditions was confirmed. We observed that colony-forming units (CFU) remained constant, with 7.7 × 10^8^ ± 2.9 × 10^8^ CFU/mL before and 9.3 × 10^8^ ± 3.2 × 10^8^ CFU/mL after 3 h of aerobic incubation. Hence, the results suggested that atmospheric oxygen levels inhibited competence development even at permissive neutral to slightly alkaline pH values, while the bacterium arrested growth but was fully viable.

The observed increase of competence levels after exchange with fresh medium motivated us to test if several sub-cultivation steps within the exponential growth phase with fresh medium could enhance the proportion of competent cells. Therefore, we sub-cultured *C. jejuni* BfR-CA-14430 in Bolton broth (final pH7.5) three times with an overall growth period of more than 60 h. We kept cells in exponential phase with maximal OD_600nm_ of 0.4, never entering stationary phase. At each step of sub-cultivation, we determined the competence level, which was maintained at 43.4 ± 3.4%. 

### 2.3. Uptake of DNA into a DNase-Resistant State Is a Main Factor for Regulation of Natural Transformation

The single cell uptake assay using covalently labelled DNA, visualizes the first step of natural transformation, i.e., DNA uptake from the environment into the cell (periplasm). In order to show its relevance for the whole process of natural transformation, we determined uptake rates and transformation rates in parallel (Table 1). Fluorescently labeled DNA and in parallel genomic *C. jejuni* DNA carrying the mutation A128G in *rps*L conferring streptomycin resistance, were added to a *C. jejuni* cell suspension. As depicted in Figure 3, these cells had been grown for 18 ± 4 h in liquid culture under several different conditions including growth to exponential vs. stationary phase, varying pH values and microaerobic vs. aerobic conditions (Figure 4). Transformation rates were calculated from the number of transformants on streptomycin-containing ColBA relative to the number of CFU under non-selective conditions. Under optimal conditions at pH 7.5 a transformation rate of 1.3 × 10^−4^ ± 2.3 × 10^−4^ was observed (Table 1). 

Consistently, bacteria incubated at pH 5.7, which did not display any DNA uptake activity, occasionally showed one resistant colony, leading to a theoretical transformation rate of ≤6.9 × 10^−9^ ± 6.2 × 10^−10^ (Table 1). The latter rate, however, was indistinguishable from a spontaneous mutation rate, since our detection limit was one resistant colony within ~7 × 10^9^ CFU (Table 1, values without DNA). At a slightly less acidic pH of 6.3 *C. jejuni* competent fraction of bacteria was 2.3 ± 0.9%, corresponding to an approximately 200-fold increased transformation rate of 1.3 × 10^−6^ ± 2.5 × 10^−6^ in comparison to pH 5.7. 

Furthermore, we correlated the changes in transformation rate relative to the fraction of bacteria with DNA foci, normalized to the “OFF-status” culture at pH 6.3. The higher the fraction of competent bacteria, displaying DNA uptake, the higher was the transformation rate. The correlation was not strictly linear, but had a maximal ratio of change in transformation rate versus change in fraction of competent cells in different experimental settings of 5.1-fold (Table 1). This suggested that the DNA uptake assay is a reliable tool to monitor natural transformation. 

### 2.4. The Concentration of CO_2_ or Carbonate and Temperature Do Not Play a Pivotal Role in Competence Regulation

During exponential growth phase, an increase in pH between pH 5.7 and pH 7.5 resulted in enhanced levels of bacteria that displayed competence (Figure 4). Under microaerobic conditions, it is expected that a more alkaline pH results in higher levels of dissolved CO_2_ and, thus, enhanced concentration of carbonate. We asked whether the amount of dissolved CO_2_ and the concentration of carbonate also triggered competence development in *C. jejuni*. Therefore, we used different CO_2_ concentrations in the gas atmosphere during growth at different pH values, keeping levels of H_2_ and O_2_ constant (Figure S3). CO_2_ concentrations varied between 0% and 50%, with intermediate concentrations of 1%, 7%, 15% and 35%. Doubling times during *C. jejuni* growth were optimal with around 1.1 h in the presence of 1% to 15% CO_2_ (Table S1), suggesting that the latter concentration range perfectly supports growth of *C. jejuni* in liquid broth culture. At higher concentrations of 35% and 50% CO_2_, mean generation times were marginally increased to 1.5 and 1.6 h, respectively (Table S1). Interestingly, even without gaseous CO_2_ *C. jejuni* was able to grow, although with 3-fold increased mean generation times (3.4 h, Table S1). For better comparison, the fraction of competent bacteria grown under standard condition at 7% CO_2_ and pH 7.5 was set to 100% for the experimental day. Although, the day-to-day variation at low concentrations of CO_2_ (0% and 1%) seemed to be large, the CO_2_ concentration itself did not correlate with the kinetics of pH-dependent switch of bacteria into the competent state (Figure S3). We also cross-checked the influence of carbonate levels on competence development in a control experiment by addition of 12 or 48 mM of sodium hydrogen carbonate to the medium and could confirm that pH-dependent switch into competent state was unaffected by the concentration of carbonate (Figure S4). 

**Figure 5:**
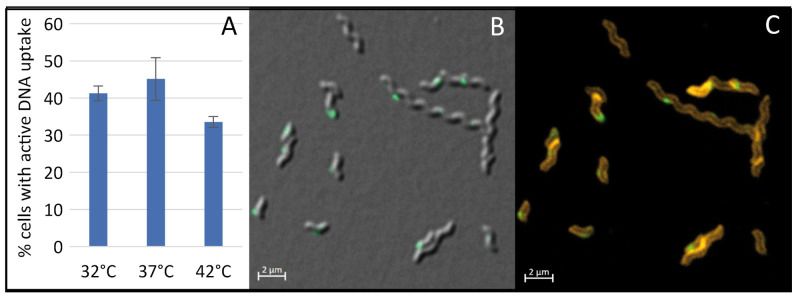
Growth temperature only marginally influences competence development in *C. jejuni*. Fraction of competent *C. jejuni* grown to exponential phase at pH 7.5 at 32 °C, 37 °C and 42 °C (**A**). Bacterial morphology and DNA uptake locations of bacteria grown at 32 °C were displayed by an overlay differential phase contrast (DIC) image with the Fluorescein channel (labelled DNA, in green) (**B**) or by an overlay image of the Fluorescein channel (labelled DNA) and the Cyanine-3 (Cy3) channel (membranes stained with the commercial dye, SynaptoRed C2) (**C**). Scale bar, 2 µm. DNA uptake was performed at 37 °C under aerobic conditions. Experiments were performed at least three times. Error bars indicate standard deviation.

Furthermore, we investigated whether temperature played a role in competence development of *C. jejuni*, as indicated by others [23]. We checked the impact of growth temperature at pH 7.5 on competence development in *C. jejuni*, choosing 32 °C as minimal growth temperature and 37 °C and 42 °C as typical host temperatures (Figure 5). The generation time of *C. jejuni* at the different temperatures was 1.2 ± 0.1 h (37 °C), 0.9 ± 0.2 h (42 °C) and 3.1 ± 0.3 h (32 °C). We observed that competence development was optimal at 37 °C with 45.1 ± 5.8% (Figure 5A). 

An increase in growth temperature to 42 °C resulted in a slight reduction of competence levels to 33.6 ± 1.4%. As expected for non-optimal growth temperature of 32 °C, the culture harbored normal length bacteria but also several elongated phenotypes (Figure 5B,C). However, competence levels were only marginally altered (41.3 ± 2.0%), if elongated cells were counted as one bacterium. Since in other bacteria, DNA uptake was localized at the cell poles or at the newly synthetized septum, we wondered, if this was also true for *C. jejuni*. We used the SynaptoRed C2 for in vivo staining of *C. jejuni* membranes in order to detect putative septa in elongated cells (Figure 5C). However, it appeared that the elongated cells did not yet harbor a septum and that localization of DNA uptake did not necessarily occur directly at the cell pole or at the next division site.

### 2.5. DNA Uptake Machinery of Competent Cells Was Shut Down at Slightly Acidic pH

**Figure 6:**
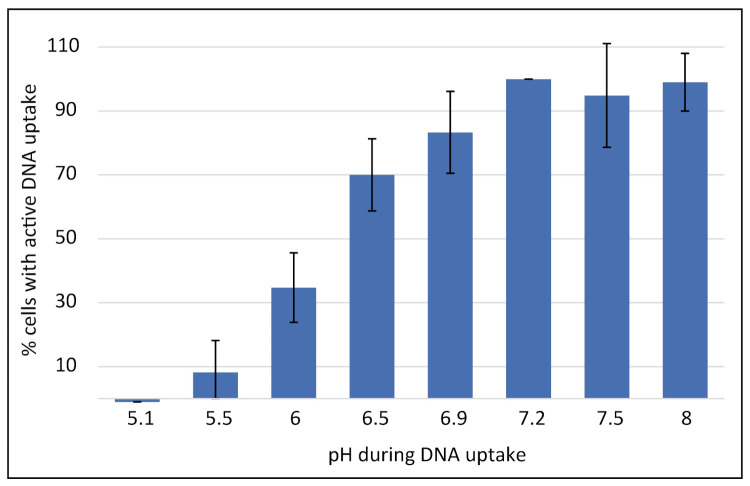
DNA uptake is completely shut down at slightly acidic pH. Competent *C. jejuni* BfR-CA-14430 bacteria were incubated with fluorescently labelled DNA in BHI titrated to the indicated pH values (±0.1) under microaerobic conditions. The fraction of bacteria showing active DNA uptake are depicted, normalized to untitrated BHI, pH 7.2 condition (set to 100%). Experiments were performed at least three times with independent cultures. Error bars indicate standard deviation. BHI, brain-heart infusion.

In order to evaluate the direct effect of pH on the DNA uptake process in already competent bacteria, multiple aliquots of a competent *C. jejuni* culture grown in BHI at pH 7.2 and displaying a competent cell fraction of 23.7 ± 6.2% and a transformation rate of 1.3 × 10^−5^ ± 8.0 × 10^−7^ in BHI at pH 7.2 were centrifuged. Subsequently, the bacteria were resuspended in pH-adjusted medium and incubated with fluorescently labelled DNA. DNA uptake was performed for 30 min at microaerobic conditions and the fraction of competent cells observed in non-titrated BHI at pH 7.2 set to 100% per experimental day. Not only competence development (see above) but also the DNA uptake process was highly pH-sensitive, with optimal transport at pH values between 7.2 and 8 (Figure 6). 

Between pH 7.2 and pH 5.5 the fraction of cells with active DNA uptake decreased to less than 10% and at pH 5.1 DNA uptake was abolished. Likewise, transformation activity at pH 5.1 was also drastically reduced to levels reaching the detection limit (2.2 × 10^−9^ ± 3.8 × 10^−9^). By pre-incubation of the bacteria with fluorescent DNA at pH 5.1 for 30 min and subsequent titration to pH 7.5, we showed that genomic DNA was stable at pH 5.1 and the pH-dependent shut down of the DNA uptake process was reversible. These cells regained DNA uptake capacity of 59.8 ± 3.2%.

## 3. Discussion

We used a single cell DNA uptake assay to monitor DNA uptake during natural transformation in *C. jejuni*. As known for *H. pylori,* this assay visualizes uptake of fluorescently labelled DNA into a DNase-resistant state, namely the periplasm [22,32], which is the first step of natural transformation. In *C. jejuni* the fraction of cells with active DNA uptake correlated with transformation rates (Table 1), was energy-dependent and limited to uptake of genomic DNA of *C. jejuni* with the methylated RATTY motif [26], suggesting that the assay was an excellent tool to decipher parameters for regulation of competence development and of the DNA uptake process itself.

*C. jejuni* grows at temperatures above 30 °C [35]. In cattle, pigs and other warm-blooded animals, including the human being, the typical temperature is around 37 °C, while in birds *C. jejuni* thrives at an elevated temperature of 42 °C. *C. jejuni* is a fastidious bacterium with special needs for growth and survival [36]. pH was revealed as major regulating parameter for competence development and DNA uptake itself. Using the classical approach with a chloramphenicol resistance marker, it was previously reported that natural transformation of *C. jejuni* efficiently occurred above pH 7 [23]. Wilson and colleagues described an effect of CO_2_ on transformation rates [24]. Using different carbonate concentrations or CO_2_ levels, with highly controlled pH values we could reveal that CO_2_ or carbonate levels did not affect competence in *C. jejuni* but that pH ≥ 7 was a prerequisite for efficient natural transformation activity. At pH 5 competence development and DNA uptake activity in *C. jejuni* was completely shut down.

Under non-growing conditions, i.e., at atmospheric conditions, *C. jejuni* ceased competence development even at permissive pH without losing viability. This is in contrast to *H. pylori* where oxidative stress was a stimulating factor for competence development [22]. However, when we measured the regulation of functional DNA uptake complexes, competent bacteria even showed slightly enhanced DNA uptake under aerobic conditions (Figure S1). In *C. coli* natural transformation of point mutation resistances was also not reduced in an aerobic atmosphere, after the cells had been grown to exponential phase under microaerobic conditions [37]. Now that our study enabled us to dissect competence development and the DNA uptake process in *C. jejuni*, the data suggest that outside the host *Campylobacter* would not be able to develop competence. However, if competent cells are shedded from the host, these bacteria might still be able to undergo genetic exchange.

At pH 7.5, and under microaerobic conditions a transformation rate of 1.3 × 10^−4^ ± 2.3 × 10^−4^ and around 50% cells with active DNA uptake were observed. Hence, one out of 4000 cells with active DNA uptake successfully recombined the genomic *rpsL* resistance marker into the chromosome and was able to grow on streptomycin-containing agar plates. Several steps of sub-cultivation in fresh medium did not further enhance competence development, confirming that bacteria stay bimodal and do not switch completely into the competent state. Variation of pH and incubation at aerobic atmosphere drastically influenced both the fraction of competent *C. jejuni* and the number of transformants. This demonstrated that the single cell assay was a suitable tool for direct monitoring of natural transformation capacity. It also implicated that regulation of natural transformation in *C. jejuni* primarily occurred at the level of regulation of the first step of DNA uptake, as observed for *H. pylori* [21,22]. Consecutive steps of natural transformation, like DNA uptake into the cytoplasm or homologous recombination appeared to be quite stable under our tested conditions, however, limiting resistance marker expression.

Growth but not cell division seemed to be important for competence development (Figure 4 and Figure 5). In stationary growth phase, DNA uptake capacity was slightly reduced. Absence of CO_2_ did support growth of *C. jejuni* under our conditions. The inoculum of 3 × 10^−7^ and 2.5 × 10^−5^, corresponding to only 1500 to 1.25 × 10^5^ bacteria per mL (assuming that OD_600_ of 0.2 corresponds to 10^9^ cell counts per ml [38]) guaranteed that respiration of *C. jejuni* was insufficient to significantly elevate the CO_2_ concentration in the medium. Growth at zero CO_2_ or at limiting growth temperature of 32 °C led to prolonged generation times and potential elongated cell morphology, but did not drastically affect competence development. Hence, neither the frequency of cell division nor the length of the generation time seemed to be signals for competence development. This was consistent with the finding that localization of DNA uptake in *C. jejuni*, as observed in *H. pylori* [22], did not strictly occur at the cell poles, as was previously shown for *B. subtilis* [39,40]. 

For colonization of the avian gut, *C. jejuni* has to first passage through the esophagus into the crop. The pH in the crop is largely influenced by the ingested food. It has been mostly measured to range between 4 and 6, with maximal values reported below 7 [41]. The pH decreases to around 3 to 4 in the gizzard due to gastric juice secretion. Our data suggest that *C. jejuni* is non-competent within passage through these parts of the avian digestive system. Once reaching the intestine, pH rises to 6–7.5, with maximal pH values of 7.9 [41] and competence might rapidly develop. Thus, the intestine is suggested to be the location with extensive genetic exchange between different *Campylobacter* spp. strains harboring DNA with the methylated DNA uptake motif. Shed competent *C. jejuni* are still able to take up DNA but will lose competence with time outside the host due to the inhibitory effect of atmospheric oxygen. We propose that *C. jejuni* increases genetic diversity at its multiplication site in order to pre-adapt to unfavorable conditions outside the host and/or for efficient host switch.

## 4. Materials and Methods

### 4.1. Strains and Growth Conditions

*C. jejuni* field strain BfR-CA-14430, isolated from chicken and previously sequenced [42] and reference strain *C. jejuni* 81-176 were used. Strains were stored at −80 °C in cryocultures (MAST Group Ltd., Bootle, UK). Cells were cultured either on Columbia blood agar plates (ColBA, Oxoid, Thermo Fisher Scientific Inc., Waltham, MA, USA), supplemented with 5% defibrinated sheep blood (Oxoid, Thermo Fisher Scientific Inc, Waltham, MA, USA), in brain heart infusion (BHI, Oxoid, Thermo Fisher Scientific Inc, Waltham, MA, USA) or in Bolton broth basis without selective supplements (Oxoid, Thermo Fisher Scientific, Waltham, MA, USA). The cultivation on ColBA was performed at 37 °C under microaerobic conditions (5% O_2_, 10% CO_2_, rest N_2_) using a microaerobic incubator (Binder, Tuttlingen, Germany). For the transformation experiments, selection of transformants was performed on ColBA supplemented with 20 µg/mL of streptomycin (Sigma-Aldrich, Steinheim, Germany). 

Liquid cultures were incubated in gas replacement jars (Oxoid Anaerobia System, Thermo Fisher Scientific Inc., Waltham, MA, USA), filled with the appropriate gas mixtures (Air Liquide, Paris, France) under shaking at 140 rpm at 37 °C. All the gas mixtures had 3.5% H_2_ and 6% O_2_ and only differed in the concentration of CO_2_ (and respective N_2_). Four gas cylinders were used: 0% CO_2_, 1% CO_2_, 7% CO_2_ and 50% CO_2_. Jars were evacuated to −70 kPa using a vacuum pump (KNF Neuberger GmbH, Freiburg im Breisgau, Germany) and the appropriate gas was filled into the jar. Evacuation to −70 kPa and refilling led to 70% exchange of atmosphere. The refilling process was done twice. To achieve 15% CO_2_, the jar was first filled with the gas mixture containing 50% CO_2_ and subsequently with 7% CO_2_ (1st filling: 0.7 × 50% CO_2_ = 35% CO_2;_ 2nd filling: 0.7 × 7% CO_2_ + 0.3 × 35% CO_2_ = 15.4%). For 35% CO_2_, the jar was filled with 0% CO_2_ in the first refilling process and, thereafter, with 50% CO_2_ (1st filling: only O_2_ was decreased and H_2_ increased; 2nd filling: 0.7 × 50% CO_2_ = 35%).

pH of media also depends on the amount of dissolved CO_2_ and, thus, on the amount of gaseous CO_2_. Hence, opening the jar will decrease CO_2_, thereby, increasing pH. We checked that during the first 10 min after opening the jar the pH only marginally increased (Figure S5) and that handling of the bacterial suspensions and pH measurements were acceptable within this time period. 

### 4.2. DNA Uptake Assay

Genomic DNA of *C. jejuni* was extracted using the PureLink Genomic DNA Kit (Thermo Fisher Scientific Inc, Waltham, MA, USA) according to manufacturer’s instructions. Non-methylated λ-DNA (Thermo Fisher Scientific Inc., Waltham, MA, USA) and extracted genomic *C. jejuni* DNA were covalently labelled with fluorescein in a 1:1 (volume:weight) ratio of Label IT reagent to nucleic acid using the Mirus Label IT Fluorescein kit (Mirus Bio LLC, Madison, WI, USA).

Cells were streaked out from a −80 °C stock culture and sub-cultured on ColBA at 37 °C in an incubator (Binder, Tuttlingen, Germany) under microaerobic conditions (5% O_2_, 10% CO_2_, rest N_2_) for 18 ± 4 h. Cells from ColBA were resuspended in BHI or Bolton broth at an initial optical density at 600 nm (OD_600_) of 0.3 and pre-cultured for 7 ± 2 h hours using gas jars. Subsequently, cells were sub-cultured in fresh BHI or Bolton if indicated pH was adjusted with sodium hydroxide (NaOH) or hydrochloric acid (HCl). An appropriate initial OD_600_ to obtain a maximal OD_600_ of 0.5 for “exponential phase” cultures and an OD_600_ between 1 and 1.5 for “stationary phase” cultures, after 18 ± 4 h was used. Over all conditions the initial OD_600_ ranged between 0.007 and 1 × 10^−6^. Generation times varied between 1 h and 5 h. This assay setup in which cells were grown in medium with pH change before 18 ± 4 h growth period was called “long-term pH”. For the assay setup “short-term pH” cells were grown for 18 ± 4 h, then the jar was opened, pH was measured (Mettler Toledo, Columbus, OH, USA), and adjusted by adding a suitable volume of sodium hydroxide (NaOH) or hydrochloric acid (HCl). For condition “fresh medium”, cultures were 3 to 5-fold diluted with fresh Bolton broth in order to reach an OD_600_ of 0.05. Indicated pH values are pH ± 0.1. Cells were incubated for another 3 h under microaerobic conditions (3.5% H_2_, 6% O_2_, 7% CO_2_, rest N_2_) or if indicated under aerobic conditions at 37 °C and shaking at 140 rpm. Afterwards cells were harvested by centrifugation for 5 min at 16,000× *g* and resuspended in 100 µL BHI. If indicated, pH was changed with NaOH or HCl. For deviation of pH between aerobic and microaerobic conditions before and after growth see Figure S2. 

For the DNA uptake assay, 1 µL labelled gDNA of *C. jejuni* (100 ng/µL) and as a control the same amount of labelled λ-DNA (Thermo Fisher Scientific Inc., Waltham, MA, USA) were added to the cells. Incubation was performed for 30 min at 37 °C under microaerobic (Figure 6, Figure S3 and Figure S4) or aerobic conditions (Figure 1, Figure 2, Figure 4 and Figure 5). The cell suspension was centrifuged for 5 min at 16,000× *g*, resuspended in 15 µL BHI (or Bolton broth) and incubated for 5 min at 37 °C after addition of 1 µL DNase (10 U) (Roche, Rotkreuz, Schweiz). If appropriate cells were stained by adding 0.4 µL 1 mg/mL SynaptoRed C2 (Sigma-Aldrich, Steinheim, Germany) to the cell suspension. Cells were immobilized on 1.5% agarose surface. For evaluation of competence status cells were analyzed using the fluorescence microscope Zeiss Axio Observer Z1 with differential phase contrast (DIC), a metal halide light source (HXP120C) and a plan apochromatic 63 ×/1.4 objective. For detection of fluorescein-labelled DNA a filter set with excitation at 470 ± 20 nm and emission at 525 ± 25 nm and for detection of SynaptoRed staining a filter set with excitation at 550 ± 12 nm and emission at 605 ± 35 nm was used. Images were taken with a 12-bit monochromatic AxioCam MRm camera. Cells harboring at least one fluorescent focus were considered competent. For each condition at least 750 cells were counted. 

To analyze if DNA uptake requires proton motive force, *C. jejuni* BfR-CA-14430 was preincubated for 10 min with 250 µM CCCP before DNA uptake was performed by incubation for 30 min at 37 °C.

### 4.3. Transformation Assay

Cells were grown as described for the DNA uptake assay. Cells were incubated for 1 h under microaerobic conditions (5% O_2_, 10% CO_2_, rest N_2_) at 37 °C with 100 ng of *C. jejuni* gDNA of strain BfR-CA-14430-strep containing the point mutation A128G in *rpsL*, conferring streptomycin resistance. Then, cells were centrifuged for 5 min at 16,000× *g* and 1 µL DNase [10 U/µL] was added. For chromosomal integration and expression of the streptomycin resistance marker, the suspension was incubated for another 3 h under microaerobic conditions (5% O_2_, 10% CO_2_, rest N_2_) at 37 °C and a serial dilution of the cells was plated onto ColBA for CFU determination as well as on ColBA containing 20 µg/mL streptomycin for quantification of transformants. Agar plates were incubated for 2 days under microaerobic atmosphere (5% O_2_, 10% CO_2_, rest N_2_) at 37 °C. Transformation rate was calculated as the ratio of transformants per CFU.

## 5. Conclusions

The single cell assay enabled us to dissect competence development and DNA uptake in *C. jejuni*. Natural transformation in *C. jejuni* was strongly regulated by pH and oxidative stress was an inhibitory factor for competence development but not DNA uptake itself. At slightly acidic conditions, natural transformation activity was shut down. The data suggest that *C. jejuni* most probably increases genetic diversity at its multiplication site in the host intestine in order to prepare the pathogen for survival in the environment and for host switch. 

## Figures and Tables

**Table 1:** Transformation rates of *C. jejuni* correlate with the fraction of competent cells, harboring at least one fluorescent DNA focus.

Condition	Transformation Rate ± SD	Fraction Competent Cells ± SD (%)	Change in Transformation Rate Compared to pH 6.3	Change in Fraction of Competent Cells Relative to pH 6.3	Ratio Change Transformation Rate vs. Change in Fraction Competent Cells
pH 5.7	≤6.9 × 10^−9^ ± 6.2 × 10^−10^	0	≤0.0053-fold	∞	n.a.
pH 6.3	1.3 × 10^−6^ ± 2.5 × 10^−6^	2.3 ± 0.9	1.0-fold	1.0-fold	1.0
pH 6.3 +3 h at pH 7.6	1.4 × 10^−5^ ± 7.8 × 10^−6^	12.2 ± 3.5	10.8-fold	5.3-fold	2.0
pH 6.3 +3 h with fresh medium	2.9 × 10^−5^ ± 2.1 × 10^−5^	33.9 ± 5.0	22.3-fold	14.7-fold	1.5
pH 6.3 +3 h aerobic at pH 7.9	1.3 × 10^−6^ ± 6.4 × 10^−7^	0.8 ± 0.4	1.0-fold	0.3-fold	2.9
pH 6.3 +3 h aerobic with fresh medium	3.7 × 10^−6^ ± 1.2 × 10^−5^	3.4 ± 1.1	2.8-fold	1.5-fold	1.9
pH 7.5	1.3 × 10^−4^ ± 2.3 × 10^−4^	45.1 ± 5.8	100-fold	19.6-fold	5.1
pH 7.5 without DNA	<6.4 × 10^−9^ ± 2.5 × 10^−9^	0	<0.0049-fold	∞	n. a.
pH 7.3 (stat phase)	6.6 × 10^−6^ ± 4.3 × 10^−6^	3.5 ± 2.1	5.1-fold	1.5-fold	3.3
pH 7.3 (stat phase) without DNA	<6.5 × 10^−9^ ± 8.0 × 10^−10^	0	<0.005-fold	∞	n.a.

After growth in Bolton broth at different pH values for 18 ± 4 h, bacteria were transformed with either genomic *C. jejuni* DNA carrying the streptomycin resistance marker A128G in the ribosomal *rpsL* gene or with fluorescent DNA in parallel. Number of transformants relative to CFU and fraction of competent cells identified by the presence of fluorescent DNaseI resistant foci were compared. Conditions are detailed in Figure 4. For estimation of spontaneous mutation rate, controls without DNA were included. Experiments were performed at least three times; ∞, infinite; n.a., not applicable; <, no transformant detected within indicated detection limit.

## Data Availability

Data is available in the manuscript and in the Supplementary Materials.

## Supplementary Materials

The following are available online at https://www.mdpi.com/article/10.3390/ijms221810169/s1, Figure S1: DNA uptake is favored under aerobic compared to microaerobic conditions, Figure S2: pH variation of pH-adjusted growth medium before and after *C. jejuni* growth into exponential phase, Figure S3: pH-dependent competence development in *C. jejuni* BfR-CA-14430 is independent of CO_2_ concentration, Figure S4: Competence development depends on pH but not on sodium hydrogen carbonate, Figure S5: pH change of Bolton broth as a function of time after opening the jar, Table S1: Generation time and final pH of BfR-CA-14430 grown under different conditions.
