# Supplementary material for: “Take It or Leave It”—Factors Regulating Competence Development and DNA Uptake in Campylobacter jejuni
Source: Int J Mol Sci. 2021 Sep 21;22(18):10169. doi: 10.3390/ijms221810169 (PMC8468864; doi:10.3390/ijms221810169)
Supplement: Supplementary file 1 [file ijms-22-10169-s001.zip › ijms-1365279-supplementary.pdf]

Table S1: Generation time and final pH of BfR-CA-14430 grown under different conditions as shown in Figures 4, 5 and S3
Mean generation time for CO_2 conditions was calculated for each experiment for pH range of 6.5-8; nd, not determined.

condition	experiment	generation time	final pH	mean generation time per condition	SD generation time per condition
7% CO_2	day 1	1.125	6.28	1.136	0.10183388
7% CO_2	day 1	1.119	6.52		
7% CO_2	day 1	1.110	6.62		
7% CO_2	day 1	1.140	7.01		
7% CO_2	day 1	1.129	7.08		
7% CO_2	day 2	1.244	6.81		
7% CO_2	day 2	1.214	6.88		
7% CO_2	day 2	1.246	7.12		
7% CO_2	day 2	1.205	7.24		
7% CO_2	day 2	1.298	7.36		
7% CO_2	day 2	1.263	7.42		
7% CO_2	day 2	1.299	7.51		
7% CO_2	day 2	1.291	7.60		
7% CO_2	day 2	1.347	7.71		
7% CO_2	day 3	1.057	7.27		
7% CO_2	day 3	1.064	7.43		
7% CO_2	day 3	1.065	7.55		
7% CO_2	day 3	1.086	7.30		
7% CO_2	day 3	1.046	7.45		
7% CO_2	day 3	1.063	7.57		
7% CO_2	day 3	1.032	6.64		
7% CO_2	day 3	1.026	7.10		
7% CO_2	day 3	1.013	7.29		
7% CO_2	day 3	1.037	7.56		
7% CO_2	day 4	1.203	6.67		
7% CO_2	day 4	1.219	7.30		
7% CO_2	day 4	1.240	7.55		
7% CO_2	day 4	nd	7.68		
7% CO_2	day 5	1.088	6.63		
7% CO_2	day 5	1.039	7.01		
7% CO_2	day 5	1.036	7.21		
7% CO_2	day 5	1.061	7.48		
7% CO_2	day 6	0.971	7.45		
7% CO_2	day 7	1.100	7.52		
0% CO_2	day 4	3.411	7.11	3.352	0.68912011
0% CO_2	day 4	3.144	7.30		
0% CO_2	day 6	2.523	7.70		
0% CO_2	day 7	4.667	6.67		
0% CO_2	day 7	3.671	7.25		
0% CO_2	day 7	3.207	7.56		
0% CO_2	day 7	2.840	7.81		
0% CO_2	day 7	5.597	8.23		
1% CO_2	day 2	1.025	7.12	1.122	0.11010026
1% CO_2	day 2	1.052	6.91		
1% CO_2	day 2	1.082	6.56		
1% CO_2	day 2	1.045	7.21		
1% CO_2	day 2	1.034	7.41		

1% CO_2	day 2	1.022	7.65		
1% CO_2	day 2	1.037	7.84		
1% CO_2	day 2	1.077	7.95		
1% CO_2	day 2	1.179	8.11		
1% CO_2	day 4	1.242	7.11		
1% CO_2	day 4	1.251	7.27		
1% CO_2	day 4	1.252	7.60		
1% CO_2	day 4	1.370	7.87		
1% CO_2	day 5	1.032	7.08		
1% CO_2	day 5	1.063	7.28		
1% CO_2	day 5	1.138	7.57		
1% CO_2	day 5	1.230	7.88		
15% CO_2	day 5	1.180	6.39	1.141	0.027423
15% CO_2	day 5	1.138	6.90		
15% CO_2	day 5	1.116	7.16		
15% CO_2	day 5	1.130	7.42		
35% CO_2	day 4	1.567	6.14	1.514	0.11842482
35% CO_2	day 4	1.379	6.73		
35% CO_2	day 4	1.598	6.93		
50% CO_2	day 4	1.629	6.04	1.558	0.327892
50% CO_2	day 4	1.427	6.72		
50% CO_2	day 4	2.143	6.93		
50% CO_2	day 5	1.278	6.02		
50% CO_2	day 5	1.255	6.78		
50% CO_2	day 5	1.613	6.98		
50% CO_2	day 5	nd	7.14		
pH 5.7	day 21	1.482	5.75	1.929	0.4960276
pH 5.7	day 22	1.842	5.68		
pH 5.7	day 23	2.463	5.75		
pH 6.3	day 9	1.090	6.36	1.189	0.11983312
pH 6.3	day 10	1.166	6.36		
pH 6.3	day 11	1.108	6.28		
pH 6.3	day 12	1.156	6.30		
pH 6.3	day 15	1.261	6.27		
pH 6.3	day 17	1.237	6.21		
pH 6.3	day 18	1.392	6.34		
pH 6.3	day 19	1.292	6.41		
pH 6.3	day 20	1.361	6.24		
pH 6.3	day 24	1.099	6.38		
pH 6.3	day 26	1.108	6.38		
pH 6.3	day 27	0.998	6.42		
pH 7.5/37°C	day 8	1.198	7.53	1.176	0.10480859
pH 7.5/37°C	day 9	0.971	7.45		
pH 7.5/37°C	day 10	1.121	7.44		
pH 7.5/37°C	day 11	1.346	7.46		
pH 7.5/37°C	day 12	1.234	7.48		
pH 7.5/37°C	day 13	1.100	7.52		
pH 7.5/37°C	day 14	1.208	7.51		
pH 7.5/37°C	day 15	0.929	7.51		
pH 7.5/37°C	day 16	1.102	7.57		
pH 7.5/37°C	day 17	1.214	7.51		
pH 7.5/37°C	day 18	1.130	7.56		
pH 7.5/37°C	day 19	1.346	7.54		
pH 7.5/37°C	day 20	1.186	7.46		
pH 7.5/37°C	day 22	1.160	7.51		

pH 7.5/37°C	day 23	1.209	7.48		
pH 7.5/37°C	day 24	1.267	7.58		
pH 7.5/37°C	day 25	1.235	7.59		
pH 7.5/37°C	day 26	1.185	7.59		
pH 7.5/37°C	day 27	1.209	7.60		
42°C	day 12	0.891	7.51	0.916	0.14744584
42°C	day 13	0.782	7.45		
42°C	day 14	1.074	7.50		
32°C	day 9	3.038	7.49	3.141	0.31153584
32°C	day 16	3.491	7.53		
32°C	day 17	2.893	7.52		

Supplementary Figures

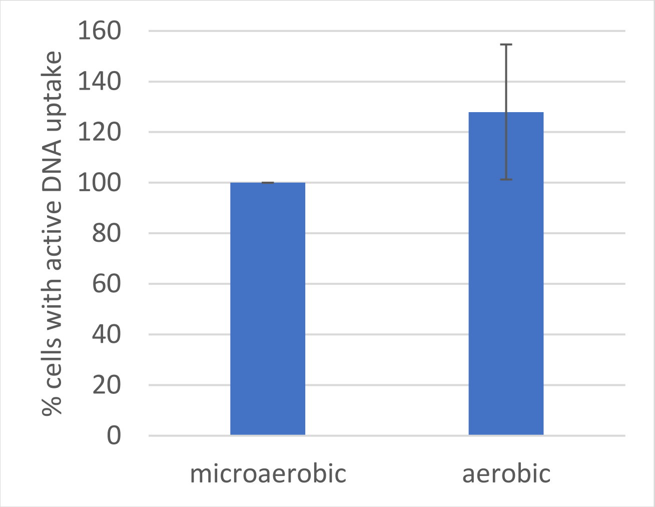

Figure S1. DNA uptake is favored under aerobic compared to microaerobic conditions. Two aliquots of competent *C. jejuni* BfR-CA-14430 were incubated with fluorescent DNA under microaerobic (set to 100%) or aerobic conditions. n = 20, error bars indicate standard deviation.

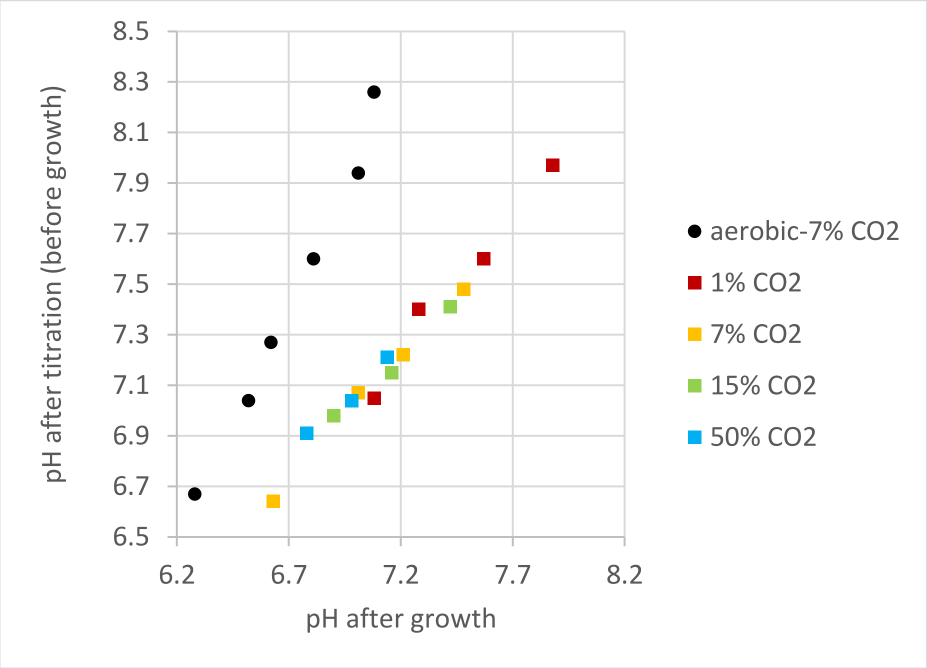

Figure S2. pH of pH-adjusted growth medium before and after growth of *C. jejuni* to exponential phase. Due to low number of bacterial cells in exponential phase, the pH of the growth medium remained constant but dependent on CO_2 concentration. Bolton broth was titrated with HCl or NaOH under aerobic conditions and incubated for at least 30 min under the indicated microaerobic conditions before pH was measured (time point "before growth", y-axis). In order to show the influence of microaerobic incubation on pH reduction, one medium was measured before growth under aerobic conditions (black dots). *C. jejuni* BfR-CA-14430 was grown until exponential growth phase and pH was measured "after growth" (x-axis). Atmospheres contained 3.5% H_2, 6% O_2 and the indicated CO_2 concentration (red, 1% CO_2, yellow, 7% CO_2, green, 15% CO_2, blue, 50% CO_2) and corresponding N_2 levels. pH after growth was measured within 5 min after opening the jar (see Fig. S5).

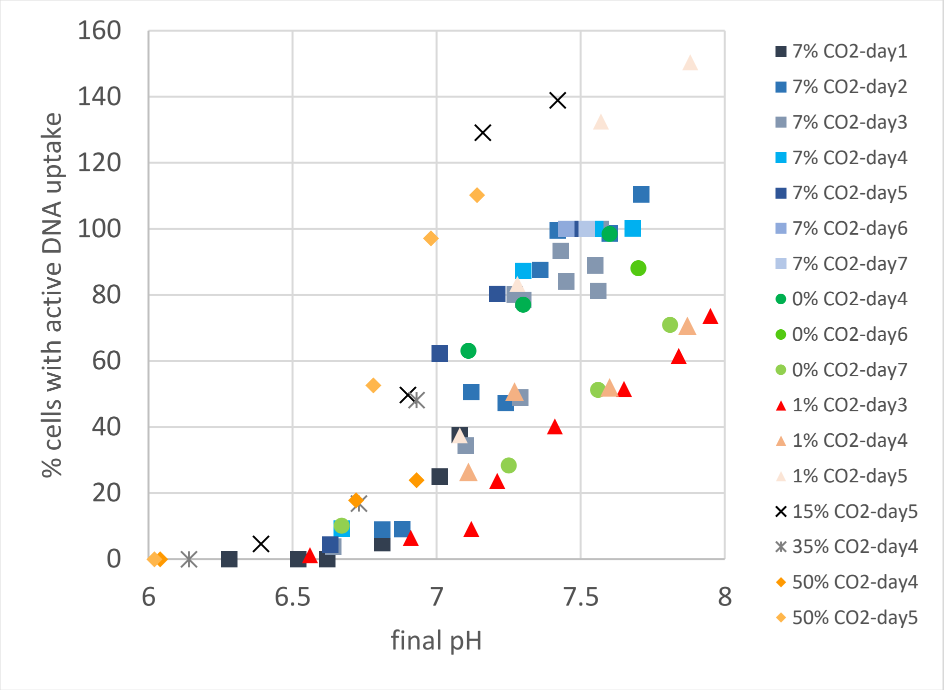

Figure S3. pH-dependent competence development in *C. jejuni* BfR-CA-14430 is independent of CO_2 concentration. Fraction of competent cells grown for 18 ± 4 h in Bolton to indicated final pH values into exponential phase were normalized per experimental day to the pH 7.5 condition at 7% CO_2 (set to 100%). DNA uptake was performed under microaerobic conditions. All tested atmospheres contained 3.5% H_2 and 6% O_2 and varying levels of CO_2 (and corresponding N_2). circles/green colors, 0% CO_2; triangles/red colors, 1% CO_2; squares/blue colors, 7% CO_2; cross/dark grey, 15% CO_2; crosses/light grey with orthogonal bar, 35% CO_2; rhombus/orange colors, 50% CO_2. Generation times for all conditions were 1.3 h ± 0.3 h, except for the atmosphere containing 0% CO_2 and 50 % CO_2,, under which generation times were enhanced up to 4.7 h or 2.1 h, respectively (see also Tab. S1).

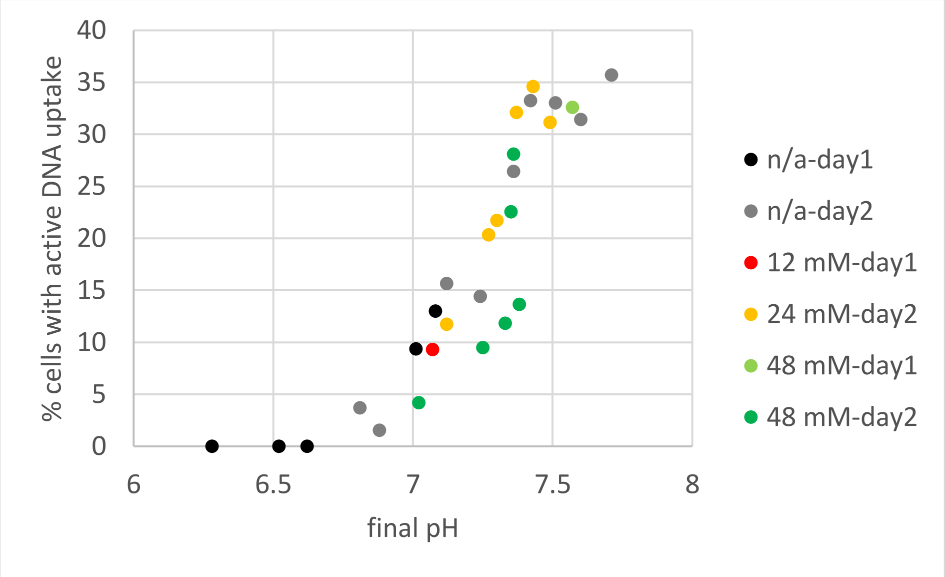

Figure S4. Competence development depends on pH but not on the concentration of sodium hydrogen carbonate ($NaHCO_3$). *C. jejuni* BfR-CA-14430 was grown for 18 ± 4 h in Bolton broth supplemented with $NaHCO_3$. n/a, no addition. Data are shown from two independent experiments (day1 and day2).

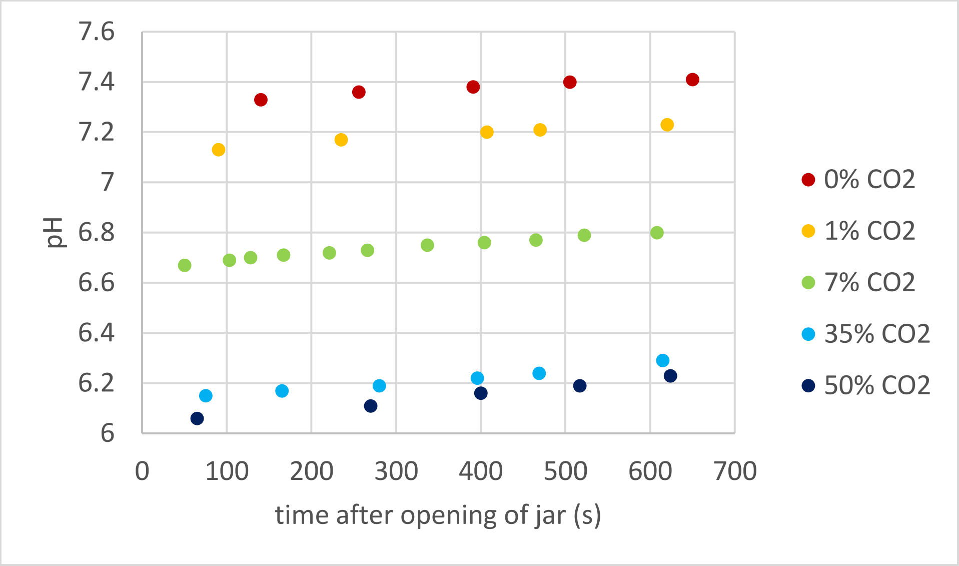

Figure S5. pH change of Bolton broth as a function of time after opening the jar. pH of Bolton was measured for around 10 min after opening the jar containing 3.5% H_2, 6% O_2 with the indicated CO_2 concentration and corresponding N_2 levels under constant agitation.
